# Oral health practices and literacy in Hungarian diabetes patients: insights from a pilot-study using a WHO-adapted questionnaire

**DOI:** 10.1186/s12903-025-05820-x

**Published:** 2025-03-26

**Authors:** Klaudia Lipták, Laura Lipták, Kata Sára Haba, Dorottya Bányai, Dániel S. Veres, Noémi Katinka Rózsa, Péter Hermann, Dániel Végh

**Affiliations:** 1 Department of Prosthodontics, Semmelweis University, Szentkirályi u. 47, Budapest, 1088 Hungary; 2 Department of Paediatric Dentistry and Orthodontics, Semmelweis University, Szentkirályi u. 47, Budapest, 1088 Hungary; 3 Department of Biophysics and Radiation Biology, Semmelweis University, Tűzoltó u. 37-47, Budapest, 1094 Hungary

**Keywords:** Diabetes, Oral health, Health education, Public health, Interdisciplinary care, Advocacy

## Abstract

**Introduction:**

This study aimed to compare the self-reported oral health status, knowledge, and habits of individuals living with diabetes mellitus and healthy controls. To achieve this, the modified version of the World Health Organization’s Oral Health Questionnaire for Adults (ANNEX 7; Google Forms) was employed for data collection.

**Methods:**

The study included 99 diabetes patients (33 with type 1 and 66 with type 2 diabetes mellitus) and 102 non-diabetic controls. Logistic regression models, adjusted for demographic factors, were applied to examine associations between oral hygiene practices, dietary habits, and the number of natural teeth.

**Results:**

Individuals with diabetes exhibited a greater frequency of oral pathological conditions. Despite 74% of people with diabetes mellitus (PwDM) brushing their teeth twice daily, they had fewer natural teeth compared to the control group (20 or more teeth: DM: 54.5%, controls: 70.6%). In our study, there is no evidence that number of teeth is dependent on HbA1c levels, and we found that neither age nor gender influenced the change in HbA1c levels. Lower interdental cleaning habits and frequent fruit consumption were identified as significant risk factors for severe tooth loss.

**Conclusion and clinical relevance:**

This study highlights significant oral health disparities in diabetes patients, particularly those with T2DM, who reported poorer oral health, more frequent denture use, and fewer natural teeth. These findings underscore the need for integrating oral health education, preventive care, and interdisciplinary collaboration into diabetes management to improve overall health outcomes.

**Supplementary Information:**

The online version contains supplementary material available at 10.1186/s12903-025-05820-x.

## Introduction

Diabetes mellitus (DM) is a metabolic disorder characterized by elevated blood glucose levels due to a lack of insulin secretion, function, or both. It is estimated that 537 million people had DM in 2021, a number that is projected to rise steadily to 643 million by 2030, and 783 million by 2045 according to the International Diabetes Federation (IDF) Diabetes Atlas [1]. The same statistics are also true for Hungary, where the most recent statistics show that DM affects one in six Hungarians and more than one third of people over 65 years [2].

Despite the growing recognition of diabetes-related oral health issues, they remain underrepresented in diabetes care. To better understand the oral health behaviors and literacy of Hungarian diabetes patients, we conducted a survey using a WHO-adapted questionnaire. This questionnaire has been previously used in international research to assess key aspects of oral hygiene habits, access to dental care, and awareness of diabetes-related oral health risks [3, 4]. The questionnaire covers several key domains: oral hygiene practices (toothbrushing frequency, flossing), oral health awareness (knowledge of diabetes-related oral health risks), dental care behaviors (regularity of dental visits, barriers to dental care), and self-perceived oral health status (gum bleeding, tooth sensitivity, dry mouth). By analyzing these aspects, our study aims to identify potential gaps in oral health education and preventive care among Hungarian diabetes patients.

Although not commonly discussed in DM care, even slightly elevated blood glucose levels can have detrimental affects on oral health, manifesting in a wide range of oral diseases and conditions [5]. More than two decades ago, periodontitis was identified as the sixth complication of DM by Harald Löe, the Director of the National Institute of Dental Research [6]. Despite this, the concept need more attention in the medical community until recently. This becomes even more important when it is recognized that periodontal disease negatively affects blood glucose levels so that periodontitis and hyperglycemia are mutually and bi-directionally related and adversely affect each other [7, 8]. In current DM management, the oral cavity is often overlooked, despite the fact that professional societies such as the Semmelweis University Diabetes Dental Research Group recommend that routine DM screening should include a referral to a dental examination [6, 9]. The two most common forms of periodontal disease are gingivitis and periodontitis, infections caused by inflammation of the soft tissues around the tooth. Gingivitis affects up to 90% of the world population [10]. It has been found that people with DM have an increased susceptibility to periodontal disease, with a risk two to three times higher than that of people without DM. In addition, research has shown a chronic and reciprocal relationship between DM and periodontal disease [11].

Chávez et al. (2001) noted that xerostomia is prevalent in up to 46% of patients with type 2 diabetes mellitus (T2DM). This condition is directly associated with reduced salivary flow and increased glucose levels in saliva, contributing to dental caries, difficulties in nutritional intake, and prosthetic use [12]. DM neuropathy often plays a role in the development of burning mouth syndrome, a painful condition that affects many DM patients, probably as a complication of long-term hyperglycemia and its impact on nerve health [13]. Patients with DM (PwDM), especially those with poorly controlled blood sugar levels, face an increased risk of implant failure and delayed healing due to heightened inflammatory responses and reduced blood circulation. In addition, a weakened immune response makes them more susceptible to infections, further complicating the healing process [14]. Furthermore, PwDM is more prone to oral infections such as candidiasis, and there is emerging evidence that DM is associated with an increased risk of oral cancers [15]. Persistent hyperglycemia and hyperinsulinemia are known to trigger abnormal cellular proliferation [16, 17].

Severe periodontal disease is the sixth most prevalent disease in the world (11.2%) [18, 19]. Given the relevant correlation between periodontal disease and DM, as well as the positive effects of maintaining good oral health to mitigate the risk of periodontal disease, it is imperative to prioritize encouraging individuals with DM to adopt proper oral hygiene practices. This includes offering regular risk assessments and dental referrals as an integral component of routine DM care [3, 20]. The implementation of this review is of paramount importance, as proficient knowledge and literacy on oral health are positively correlated with favorable oral health practices. These practices include a heightened frequency of toothbrushing, regular dental appointments, and the maintenance of good periodontal health [21, 22].

We extended an earlier study, the “Budapest Pilot Study”, in Hungary. This pilot study contributes to the global understanding by offering insights into the oral health practices and knowledge of Hungarian diabetes patients and controls. This aim of this study was to collect data on the oral health knowledge and practices of PwDM. Despite growing evidence linking DM and oral health, limited studies focus on the bidirectional relationship in specific populations, such as Hungarian patients, who may exhibit unique oral hygiene practices and risk profiles. This study aims to address this gap by providing region-specific data. Our primary goal was to gain insight into the oral health habits and education levels among PwDM.

## Methods

This study is classified as a pilot study as it represents an initial exploration of oral health behaviors and awareness among Hungarian diabetes patients using a WHO-adapted questionnaire. The primary objective was to assess feasibility, refine research methodology, and identify key trends that could inform future large-scale studies.

Our study involved 99 participants (33 with type 1 diabetes mellitus (T1DM) and 66 with T2DM) (Diabetes Descriptive plots) and 102 controls; 18 years of age or over. A total of 103 responses were collected in the diabetes group, of which 4 were excluded due to incomplete data. In the control group, 102 responses were received, with no exclusions. The survey was open to responses from October 1, 2022 to January 31, 2024 and could be accessed through a Google Survey (Google LLC, Mountain View, CA, USA) form on a variety of devices, including computers, tablets, or mobile phones. The presence of DM was our priority. The study included clinically confirmed diabetes patients, with no additional measurements conducted to determine their diabetes status. The questionnaires were distributed both in-person at diabetology outpatient clinics, where patients completed them during their visits, and online within diabetes communities. The study did not involve a predefined sample size calculation. Participation in the survey was voluntary, and certain criteria were applied to ensure data integrity, such asthe exclusion of incomplete questionnaires and multiple submissions from the same IP or email address to avoid duplicate responses, adhering to General Data Protection Regulation (GDPR) regulations for data protection. Participants created a unique identifier consisting of three letters and three numbers (e.g., ABC123) when filling out the questionnaire. This identifier facilitated data comparison. In cases where responses originated from the same IP address or email, they were reviewed for identical answers. If completely identical responses had been identified, they would have been excluded; however, no such cases were found. The survey language was Hungarian, and the questionnaire was translated accordingly to ensure clarity for all participants. Data were obtained from online sources and subsequently stored using Google Surveys (Google LLC, Mountain View, CA, USA) and Microsoft Excel (Microsoft Corporation, Redmond, WA, USA). In the study, we first compared the control group and the DM group, and then compared the responses of people with T1DM and T2DM.

### Online questionnaire

The questionnaire comprised a total of 24 inquiries and included questions essential for a national oral health surveillance (Table 1). The study protocol was based on ANNEX 7 of the World Health Organisation (WHO) survey [23]. The questionnaire was designed to ensure complete participant anonymity, as no personally identifiable information was collected. These structured questionnaires contain fundamental questions essential for national oral health surveillance. The collected data included the following variables: sex, age, place of residence, type of diabetes, number of teeth, presence of oral discomfort, use of removable dentures, oral hygiene habits, self-reported oral health status, types of oral care products used, toothpaste preference, frequency of dental visits, dietary and alcohol consumption habits, smoking status, level of primary education, potential fear of dental treatment, the type of medication used for diabetes management, and whether the participant uses a continuous glucose monitoring (CGM) sensor, therapy of diabetes mellitus (tablets or insulin), level of the last HbA1c. HbA1c represents the average blood glucose level over the past three months and is regularly monitored during diabetology outpatient visits. In this study, participants self-reported their most recent HbA1c value through a specific question in the questionnaire.

**Table 1:** Dental status and compliance

	Table 1a			Table 1b			
	Control (*N* = 102)	DM (*N* = 99)	Overall (*N* = 201)	T1DM (*N* = 33)		T2DM (*N* = 66)	Overall (*N* = 99)
**Sex**							
Female	74 (72.5%)	75 (75.8%)	149 (74.1%)	25 (75.8%)		50 (75.8%)	75 (75.8%)
Male	28 (27.5%)	23 (23.2%)	51 (25.4%)	8 (24.2%)		15 (22.7%)	23 (23.2%)
Missing data	0 (0%)	1 (1.0%)	1 (0.5%)	0 (0%)		1 (1.5%)	1 (1.0%)
**Age [years]**							
Mean (SD)	47.8 (17.2)	53.5 (18.1)	50.6 (17.8)	38.8 (16.3)		60.8 (14.1)	53.5 (18.1)
Median (IQR)	47.5 (35.3)	54.0 (26.0)	51.0 (31.0)	38.0 (24.0)		66.0 (18.8)	54.0 (26.0)
Min, Max	21.0, 80.0	10.0, 81.0	10.0, 81.0	10.0, 69.0		20.0, 81.0	10.0, 81.0
**Residence**							
City	90 (88.2%)	76 (76.8%)	166 (82.6%)	26 (78.8%)		50 (75.8%)	76 (76.8%)
Countryside	9 (8.8%)	14 (14.1%)	23 (11.4%)	4 (12.1%)		10 (15.2%)	14 (14.1%)
Suburb	3 (2.9%)	9 (9.1%)	12 (6.0%)	3 (9.1%)		6 (9.1%)	9 (9.1%)
**Education**							
Less than primary school	0 (0%)	1 (1.0%)	1 (0.5%)	1 (3.0%)		0 (0%)	1 (1.0%)
Primary school completed	4 (3.9%)	5 (5.1%)	9 (4.5%)	4 (12.1%)		1 (1.5%)	5 (5.1%)
Secondary/High school completed	31 (30.4%)	38 (38.4%)	69 (34.3%)	9 (27.3%)		29 (43.9%)	38 (38.4%)
Vocational school completed	16 (15.7%)	15 (15.2%)	31 (15.4%)	4 (12.1%)		11 (16.7%)	15 (15.2%)
College/university completed	48 (47.1%)	39 (39.4%)	87 (43.3%)	15 (45.5%)		24 (36.4%)	39 (39.4%)
Postgraduate degree	3 (2.9%)	1 (1.0%)	4 (2.0%)	0 (0%)		1 (1.5%)	1 (1.0%)
**Diabetes mellitus**							
T1DM	0 (0%)	33 (33.3%)	33 (16.4%)	33 (100%)		0 (0%)	33 (33.3%)
T2DM	0 (0%)	66 (66.7%)	66 (32.8%)	0 (0%)		66 (100%)	66 (66.7%)
Missing data	102 (100%)	0 (0%)	102 (50.7%)				
**HbA1c level [%]**							
Mean (SD)	NA	6.75 (1.14)	6.75 (1.14)	6.64 (0.734)		6.80 (1.29)	6.75 (1.14)
Median (IQR)	NA	6.80 (1.13)	6.80 (1.13)	6.70 (1.10)		6.80 (1.20)	6.80 (1.13)
Min, Max	NA	4.80, 14.0	4.80, 14.0	5.10, 8.00		4.80, 14.0	4.80, 14.0
Missing data	102 (100%)	0 (0%)	102 (50.7%)				
**Therapy**							
Both	0 (0%)	11 (11.1%)	11 (5.5%)	2 (6.1%)		9 (13.6%)	11 (11.1%)
Insulin	0 (0%)	37 (37.4%)	37 (18.4%)	28 (84.8%)		9 (13.6%)	37 (37.4%)
Nothing	0 (0%)	2 (2.0%)	2 (1.0%)	0 (0%)		2 (3.0%)	2 (2.0%)
Tablet	0 (0%)	46 (46.5%)	46 (22.9%)	3 (9.1%)		43 (65.2%)	46 (46.5%)
Missing data	102 (100%)	3 (3.0%)	105 (52.2%)	0 (0%)		3 (4.5%)	3 (3.0%)
**Sensor**							
No	0 (0%)	78 (78.8%)	78 (38.8%)	13 (39.4%)		65 (98.5%)	78 (78.8%)
Yes	0 (0%)	21 (21.2%)	21 (10.4%)	20 (60.6%)		1 (1.5%)	21 (21.2%)
Missing data	102 (100%)	0 (0%)	102 (50.7%)				
**Complaint**							
Yes	10 (9.8%)	18 (18.2%)	28 (13.9%)	22 (66.7%)		28 (42.4%)	50 (50.5%)
No	19 (18.6%)	23 (23.2%)	42 (20.9%)	11 (33.3%)		33 (50.0%)	44 (44.4%)
Don’t know	72 (70.6%)	54 (54.5%)	126 (62.7%)	0 (0%)		5 (7.6%)	5 (5.1%)
**Removable**							
Lower and upper complete dentures	1 (1.0%)	5 (5.1%)	6 (3.0%)	0 (0%)		5 (7.6%)	5 (5.1%)
Lower and upper complete dentures, Fixed dentures	0 (0%)	1 (1.0%)	1 (0.5%)				
Lower complete dentures	3 (2.9%)	0 (0%)	3 (1.5%)	0 (0%)		0 (0%)	0 (0%)
Lower complete dentures, Fixed dentures	0 (0%)	1 (1.0%)	1 (0.5%)	0 (0%)		1 (1.5%)	1 (1.0%)
Upper complete dentures	2 (2.0%)	5 (5.1%)	7 (3.5%)	2 (6.1%)		3 (4.5%)	5 (5.1%)
Upper complete dentures, Fixed dentures	1 (1.0%)	3 (3.0%)	4 (2.0%)	0 (0%)		3 (4.5%)	3 (3.0%)
I don’t wear any dentures	57 (55.9%)	52 (52.5%)	109 (54.2%)	26 (78.8%)		26 (39.4%)	52 (52.5%)
Partial dentures	6 (5.9%)	12 (12.1%)	18 (9.0%)	3 (9.1%)		9 (13.6%)	12 (12.1%)
Fixed dentures	32 (31.4%)	20 (20.2%)	52 (25.9%)	2 (6.1%)		18 (27.3%)	20 (20.2%)
**Condition teeth**							
Average	29 (28.4%)	28 (28.3%)	57 (28.4%)	6 (18.2%)		22 (33.3%)	28 (28.3%)
Good	36 (35.3%)	24 (24.2%)	60 (29.9%)	11 (33.3%)		13 (19.7%)	24 (24.2%)
Excellent	6 (5.9%)	3 (3.0%)	9 (4.5%)	2 (6.1%)		1 (1.5%)	3 (3.0%)
Very good	15 (14.7%)	9 (9.1%)	24 (11.9%)	3 (9.1%)		6 (9.1%)	9 (9.1%)
Very poor	3 (2.9%)	12 (12.1%)	15 (7.5%)	5 (15.2%)		7 (10.6%)	12 (12.1%)
Don’t know	1 (1.0%)	4 (4.0%)	5 (2.5%)	1 (3.0%)		3 (4.5%)	4 (4.0%)
Poor	12 (11.8%)	19 (19.2%)	31 (15.4%)	5 (15.2%)		14 (21.2%)	19 (19.2%)
**Condition gum**							
Average	23 (22.5%)	30 (30.3%)	53 (26.4%)	5 (15.2%)		25 (37.9%)	30 (30.3%)
Good	35 (34.3%)	23 (23.2%)	58 (28.9%)	10 (30.3%)		13 (19.7%)	23 (23.2%)
Excellent	15 (14.7%)	4 (4.0%)	19 (9.5%)	2 (6.1%)		2 (3.0%)	4 (4.0%)
Very good	10 (9.8%)	10 (10.1%)	20 (10.0%)	5 (15.2%)		5 (7.6%)	10 (10.1%)
Very poor	4 (3.9%)	11 (11.1%)	15 (7.5%)	5 (15.2%)		6 (9.1%)	11 (11.1%)
Don’t know	1 (1.0%)	4 (4.0%)	5 (2.5%)	1 (3.0%)		3 (4.5%)	4 (4.0%)
Poor	14 (13.7%)	17 (17.2%)	31 (15.4%)	5 (15.2%)		12 (18.2%)	17 (17.2%)
**Last visit**							
2 years or more but less than 5 years	9 (8.8%)	15 (15.2%)	24 (11.9%)	5 (15.2%)		10 (15.2%)	15 (15.2%)
5 years or more	5 (4.9%)	10 (10.1%)	15 (7.5%)	2 (6.1%)		8 (12.1%)	10 (10.1%)
6–12 months	32 (31.4%)	21 (21.2%)	53 (26.4%)	9 (27.3%)		12 (18.2%)	21 (21.2%)
Less than 6 months	41 (40.2%)	35 (35.4%)	76 (37.8%)	9 (27.3%)		26 (39.4%)	35 (35.4%)
More than 1 year but less than 2 years	15 (14.7%)	18 (18.2%)	33 (16.4%)	8 (24.2%)		10 (15.2%)	18 (18.2%)
**Last visit reason**							
Pain or trouble with teeth, gums or mouth	21 (20.6%)	28 (28.3%)	49 (24.4%)	10 (30.3%)		18 (27.3%)	28 (28.3%)
Treatment/ follow-up treatment	40 (39.2%)	32 (32.3%)	72 (35.8%)	8 (24.2%)		24 (36.4%)	32 (32.3%)
Consultation/advise	7 (6.9%)	10 (10.1%)	17 (8.5%)	3 (9.1%)		7 (10.6%)	10 (10.1%)
Don’t know/don’t remember	0 (0%)	3 (3.0%)	3 (1.5%)	1 (3.0%)		2 (3.0%)	3 (3.0%)
Routine check-up/treatment	34 (33.3%)	26 (26.3%)	60 (29.9%)	11 (33.3%)		15 (22.7%)	26 (26.3%)
**Fear dentist**							
Yes	42 (41.2%)	44 (44.4%)	86 (42.8%)	17 (51.5%)		27 (40.9%)	44 (44.4%)
No	60 (58.8%)	55 (55.6%)	115 (57.2%)	16 (48.5%)		39 (59.1%)	55 (55.6%)
By DM (only DM patients)							

### Statistical analysis and visualisation

Statistical analyses were performed using R software, an open-source programming language and software environment widely used for statistical computing, data analysis, and visualization (v4.3.3, R Foundation for Statistical Computing, Vienna, Austria). We used the `Table 1` (v1.4.3) package to simplify the creation of descriptive statistical tables (Data Science, Munich, Germany), and `ggplot2` (v3.5.0) to create complex and customizable visualizations (RStudio, Boston, USA) [24–26]. To interpret regression analyses, we employed the `gtsummary` (v1.7.2) package to summarize statistical models in clear tables (RStudio, Boston, USA), and `sjPlot` (v2.8.16) to visualize model results and relationships between variables (Statistical Software, Berlin, Germany) [27, 28]. Regression models were used to assess the effect of teeth cleaning frequency and DM (PwDM versus non-DM patients) on number of teeth and the effect of number of teeth and diabetes on HbA1c level. Due to the limited sample size, only the key variables of interest and the most relevant potential confounders were included as predictors. The primary focus was on comparing diabetes and non-diabetes patients, as well as examining the type of diabetes (T1DM vs. T2DM). Additionally, eating habits (fruit and rapidly absorbable carbohydrate consumption) were analyzed. Age, sex, and oral hygiene behaviors (frequency of tooth cleaning and flossing) were considered potential confounders in the analysis.

In cases where the number of teeth was the outcome variable, as the assumptions of ordinal logistic regression did not fit, simple logistic regression models (with logit link) were used pairwise to the number of tooth categories, where the reference level was always the larger number of teeth. The effect on the HbA1c level was assessed using a linear regression model. The effects were adjusted for sex and age in both models. Two-level interactions were assessed but were found non-significant (at 5%) and not relevant and were therefore excluded from the final models reported.

## Results

A total of 99 patients with DM, comprising 24 men and 75 women, and 28 men and 74 women in the control group, participated in this survey. The mean age was 53.5 years in the DM group and 47.8 years in the control group. Among PwDM, one-third had T1DM, while the majority had T2DM. Most participants had a college/university degree. Regarding diabetes management, most PwDM used oral medication or insulin therapy, and 21.2% used a sensor to control blood glucose levels. The standard deviation of HbA1c levels of DM participants was 6.75% and the median was 6.8%. Self-reported oral health varied between groups: PwDM were more likely to rate their teeth and gums as “bad” compared to controls. One-third of PwDM had seen a dentist in the past six months, slightly lower than in the control group. The most common reasons for the last visit were treatment/follow-up treatment and routine check-ups. The proportions of patients with DM and controls wearing the same type of dentures were almost the same, and with fixed dentures being the most common, whereas in complete edentulism, participants wore lower and upper removable dentures. Over half of participants reported no fear of dental procedures (Table 1a).

Twice as many T2DM patients completed the questionnaire compared to T1DM patients, with a higher mean age in the T2DM group. The median HbA1c levels were nearly identical between the two types (T1DM: 6.7%; T2DM: 6.8%). Continuous Glucose Monitors (CGMs) were predominantly used by T1DM patients (60.6%), whereas their use was minimal among T2DM patients. A CGM is a device that continuously monitors glucose levels through a subcutaneous sensor. It is advantageous because it provides real-time glucose data, helps users detect trends, and offers alerts for high or low blood sugar levels, allowing better management of diabetes and reducing the need for frequent finger-prick tests. A higher proportion of T1DM patients wore no dentures (78.8%), suggesting that their dentition was better preserved. The subjective perception of T2DM patients was worse, with 33.3% of them considering the condition of their teeth and 37.9% the condition of their gums to be “average”. There was no significant difference between the two groups in responses to the last dental visit or fear of dental procedures (Table 1b).

A total of 69.2% of the respondents (DM group, controls) brushed their teeth at least twice a day, while 30.8% brushed once or less. Dental floss was the most common interdental cleaning tool. Among PwDM, 50.5% used only a toothbrush, while 17.2% also flossed, compared to 29.4% in the control group. Regarding fluoride awareness, 19.2% of PwDM were unsure if their toothpaste contained fluoride, while 80.4% of the control group actively chose fluoride-containing toothpaste (Table 2a).

**Table 2:** Oral hygiene and habits

	Table 2a			Table 2b		
	Control (*N* = 102)	DM (*N* = 99)	Overall (*N* = 201)	T1DM (*N* = 33)	T2DM (*N* = 66)	Overall (*N* = 99)
**Frequency teeth clean**						
2–3 times a week	2 (2.0%)	3 (3.0%)	5 (2.5%)	2 (6.1%)	1 (1.5%)	3 (3.0%)
Once a month	0 (0%)	3 (3.0%)	3 (1.5%)	2 (6.1%)	1 (1.5%)	3 (3.0%)
Once a week	1 (1.0%)	3 (3.0%)	4 (2.0%)	2 (6.1%)	1 (1.5%)	3 (3.0%)
Twice or more a day	77 (75.5%)	62 (62.6%)	139 (69.2%)	15 (45.5%)	47 (71.2%)	62 (62.6%)
Once a day	22 (21.6%)	27 (27.3%)	49 (24.4%)	12 (36.4%)	15 (22.7%)	27 (27.3%)
Never	0 (0%)	1 (1.0%)	1 (0.5%)	0 (0%)	1 (1.5%)	1 (1.0%)
**Frequency teeth clean**						
At least twice a day	77 (75.5%)	62 (62.6%)	139 (69.2%)	15 (45.5%)	47 (71.2%)	62 (62.6%)
Maximum once a day	25 (24.5%)	37 (37.4%)	62 (30.8%)	18 (54.5%)	19 (28.8%)	37 (37.4%)
**Tools**						
Other	1 (1.0%)	0 (0%)	1 (0.5%)	0 (0%)	0 (0%)	0 (0%)
Toothbrush	31 (30.4%)	50 (50.5%)	81 (40.3%)	17 (51.5%)	33 (50.0%)	50 (50.5%)
Toothbrush, Charcoal	0 (0%)	1 (1.0%)	1 (0.5%)	1 (3.0%)	0 (0%)	1 (1.0%)
Toothbrush, Other	10 (9.8%)	5 (5.1%)	15 (7.5%)	1 (3.0%)	4 (6.1%)	5 (5.1%)
Toothbrush, Wooden toothpicks	6 (5.9%)	6 (6.1%)	12 (6.0%)	3 (9.1%)	3 (4.5%)	6 (6.1%)
Toothbrush, Wooden toothpicks, Charcoal	0 (0%)	1 (1.0%)	1 (0.5%)	1 (3.0%)	0 (0%)	1 (1.0%)
Toothbrush, Wooden toothpicks, Other	0 (0%)	1 (1.0%)	1 (0.5%)	1 (3.0%)	0 (0%)	1 (1.0%)
Toothbrush, Wooden toothpicks, Dentalfloss	7 (6.9%)	2 (2.0%)	9 (4.5%)	1 (3.0%)	1 (1.5%)	2 (2.0%)
Toothbrush, Wooden toothpicks, Dentalfloss, Other	1 (1.0%)	2 (2.0%)	3 (1.5%)	0 (0%)	2 (3.0%)	2 (2.0%)
Toothbrush, Wooden toothpicks, Plastic toothpicks	0 (0%)	1 (1.0%)	1 (0.5%)	0 (0%)	1 (1.5%)	1 (1.0%)
Toothbrush, Wooden toothpicks, Plastic toothpicks, Other	1 (1.0%)	0 (0%)	1 (0.5%)	0 (0%)	0 (0%)	0 (0%)
Toothbrush, Wooden toothpicks, Plastic toothpicks, Dentalfloss, Charcoal	1 (1.0%)	0 (0%)	1 (0.5%)	0 (0%)	0 (0%)	0 (0%)
Toothbrush, Wooden toothpicks, Chewstick/miswak	0 (0%)	1 (1.0%)	1 (0.5%)	0 (0%)	1 (1.5%)	1 (1.0%)
Toothbrush, Dentalfloss	30 (29.4%)	17 (17.2%)	47 (23.4%)	6 (18.2%)	11 (16.7%)	17 (17.2%)
Toothbrush, Dentalfloss, Charcoal	2 (2.0%)	0 (0%)	2 (1.0%)	0 (0%)	0 (0%)	0 (0%)
Toothbrush, Dentalfloss, Other	9 (8.8%)	3 (3.0%)	12 (6.0%)	0 (0%)	3 (4.5%)	3 (3.0%)
Toothbrush, Plastic toothpicks	0 (0%)	2 (2.0%)	2 (1.0%)	0 (0%)	2 (3.0%)	2 (2.0%)
Toothbrush, Plastic toothpicks, Dentalfloss	2 (2.0%)	4 (4.0%)	6 (3.0%)	2 (6.1%)	2 (3.0%)	4 (4.0%)
Toothbrush, Plastic toothpicks, Dentalfloss, Charcoal	0 (0%)	1 (1.0%)	1 (0.5%)	0 (0%)	1 (1.5%)	1 (1.0%)
Toothbrush, Chewstick/miswak	1 (1.0%)	0 (0%)	1 (0.5%)	0 (0%)	0 (0%)	0 (0%)
Dentalfloss	0 (0%)	1 (1.0%)	1 (0.5%)	0 (0%)	1 (1.5%)	1 (1.0%)
Missing data	0 (0%)	1 (1.0%)	1 (0.5%)	0 (0%)	1 (1.5%)	1 (1.0%)
**Toothbrush**						
Toothbrush	101 (99.0%)	98 (99.0%)	199 (99.0%)	33 (100%)	65 (98.5%)	98 (99.0%)
None	1 (1.0%)	1 (1.0%)	2 (1.0%)	0 (0%)	1 (1.5%)	1 (1.0%)
**Dentalfloss**						
Dentalfloss	52 (51.0%)	21 (21.2%)	73 (36.3%)	0 (0%)	21 (31.8%)	21 (21.2%)
None	50 (49.0%)	78 (78.8%)	128 (63.7%)	33 (100%)	45 (68.2%)	78 (78.8%)
**Other**						
Yes	22 (21.6%)	20 (20.2%)	42 (20.9%)	0 (0%)	20 (30.3%)	20 (20.2%)
None	80 (78.4%)	79 (79.8%)	159 (79.1%)	33 (100%)	46 (69.7%)	79 (79.8%)
**Toothpaste**						
Yes	102 (100%)	96 (97.0%)	198 (98.5%)	33 (100%)	63 (95.5%)	96 (97.0%)
No	0 (0%)	3 (3.0%)	3 (1.5%)	0 (0%)	3 (4.5%)	3 (3.0%)
**Fluoride**						
Yes	82 (80.4%)	66 (66.7%)	148 (73.6%)	23 (69.7%)	43 (65.2%)	66 (66.7%)
No	11 (10.8%)	14 (14.1%)	25 (12.4%)	5 (15.2%)	9 (13.6%)	14 (14.1%)
Don’t know	9 (8.8%)	19 (19.2%)	28 (13.9%)	5 (15.2%)	14 (21.2%)	19 (19.2%)
	By diabetes (only diabetic patients)					
Table 2c	**At least twice a day** ** (N = 139)**	**Maximum once a day** ** (N = 62)**	**Overall** ** (N = 201)**			
**Teeth**						
1–9 teeth	22 (15.8%)	11 (17.7%)	33 (16.4%)			
10–19 teeth	26 (18.7%)	16 (25.8%)	42 (20.9%)			
20 teeth or more	91 (65.5%)	35 (56.5%)	126 (62.7%)			
Table 2d	**1–9 teeth** ** (N = 22)**	**10–19 teeth** ** (N = 23)**	**20 teeth or more** ** (N = 54)**	**Overall** ** (N = 99)**		
**HbA1c level [%]**						
Mean (SD)	6.81 (0.950)	7.06 (1.72)	6.59 (0.858)	6.75 (1.14)		
Median (IQR)	6.90 (1.05)	6.80 (1.44)	6.59 (1.18)	6.80 (1.13)		
Min, Max	4.80, 9.10	5.40, 14.0	4.80, 8.10	4.80, 14.0		

The responses show 71.2% of T2DM patients brushed their teeth at least twice daily, compared to 45.5% of T1DM patients. The use of oral care products and fluoride toothpaste was similar in both groups (Table 2b).

Despite more frequent brushing, PwDM had fewer teeth than the control group. However, the proportion of respondents with 20 or more teeth was not significantly lower in PwDM (54.5% vs. 70.6% in controls) (Table 2c).

Examining the association between HbA1c and the number of teeth, we found no evidence that HbA1c levels are dependent on number of teeth, considering gender and age, and we found that neither age nor gender explained (affected) the variation in HbA1c levels significantly (Table 2d). Although not statistically significant, the lower number of natural teeth in PwDM may indicate potential long-term complications, highlighting the importance of early dental interventions [29]. The relationship between HbA1c levels and dental prostheses was not analyzed, as prosthesis use reflects patient compliance rather than a determinant of diabetes.

To evaluate the impact of dental floss use and diet (fruit and rapidly absorbable carbohydrate consumption) on dental health, logistic regression models were applied, adjusting for age and sex. As ordinal logistic regression was unsuitable, pairwise logistic regression was used, with the reference always being the greater number of teeth. Results showed that individuals consuming more fruit had 3.8 times higher odds of having 1–9 teeth (vs. 10–19), independent of age, sex, tooth cleaning, flossing, and other carbohydrate intake (Table 3/a). For fruit consumption, the odds ratio of 3.81 was considered high. However, the effects of excessive consumption (eating too much) and group classification (diabetes vs. controls) were not statistically significant, though they may still hold clinical relevance. A similar association was observed when comparing 1–9 teeth to > 20 teeth (Table 3/b). Fruit consumption proved to be high in effect, while excessive consumption (eating too much) was negligible. The impact of the group variable (diabetes vs. non-diabetes) was moderate—borderline in significance. Additionally, in this comparison, the odds were about 2.94 times higher among those who did floss and approximately 1.1 times greater for each year of growth (adjusted for DM, sex, or consumption of other rapidly absorbable carbohydrates). (Table 3/c). In this case, these effects (fruit consumption, eating too much, group) do not appear to be clinically relevant.

**Table 3:** Impact of diet and flossing on dental health across tooth categories

	Table 3/a			Table 3/b			Table 3/c		
**Characteristic**	OR*	95% CI**	**p-value**	OR*	95% CI**	**p-value**	OR*	95% CI**	**p-value**
**Frequency teeth clean**			0.74			0.31			0.19
*At least twice a day*	—	—		—	—		—	—	
*Maximum once a day*	0.82	0.25, 2.63		1.81	0.58, 5.80		1.88	0.73, 4.93	
**Sex**			0.59			0.16			0.89
*Female*	—	—		—	—		—	—	
*Male*	0.69	0.17, 2.61		0.40	0.10, 1.43		01.07	0.38, 2.96	
**Age [years]**	01.02	0.98, 1.06	0.39	1.10	1.06, 1.15	**< 0.001**	01.09	1.06, 1.13	**< 0.001**
**Dentalfloss**			0.52			**0.050**			0.48
*Dentalfloss*	—	—		—	—		—	—	
*None*	1.47	0.45, 4.97		2.94	1.00, 9.44		1.39	0.56, 3.55	
**Fruits eating**			**0.020**			**0.023**			0.41
*Less*	—	—		—	—		—	—	
*More*	3.81	1.23, 13.4		3.82	1.19, 14.2		0.68	0.27, 1.69	
**Eating too much**			0.25			0.84			0.36
*No*	—	—		—	—		—	—	
*Yes*	1.86	0.65, 5.49		1.11	0.40, 3.12		0.67	0.28, 1.57	
**Group**			0.31			0.27			0.88
*Control*	—	—		—	—		—	—	
*Diabetes mellitus*	1.72	0.60, 5.09		1.77	0.64, 5.06		0.94	0.39, 2.20	

* OR = Odds Ratio

** CI = Confidence Interval

**Fig. 1:**
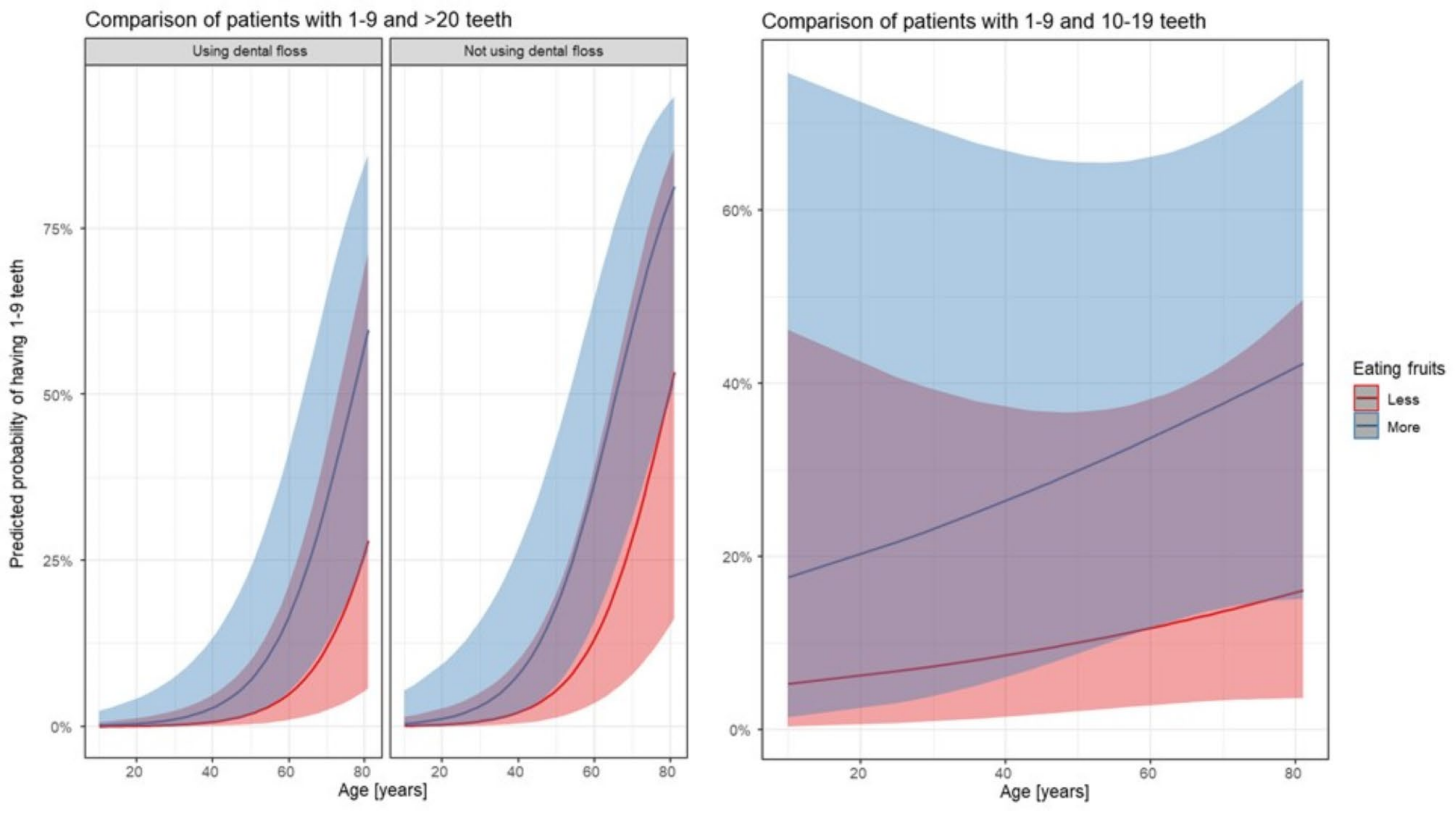
Prediction plots: Predicted tooth retention by age, flossing, and fruit consumption

Figure 1 shows the predicted probability of having 1–9 teeth based on age, flossing habits, and fruit consumption. Although the effects were not statistically significant in this sample, the observed trends suggest potential clinical relevance. These findings align with previous research on the impact of diet and oral hygiene on dental health, especially in diabetes patients [30, 31]. The lack of significance may be due to the limited sample size and cross-sectional design, highlighting the need for larger studies.

Although some results were not statistically significant, this does not rule out a potential association. Given the pilot nature of this study, a larger sample size may be required to detect meaningful these effects.

## Discussion

Our findings align with previous research indicating higher rates of tooth loss among diabetes patients globally [29]. However, the relatively lower interdental cleaning habits observed in the Hungarian cohort suggest regional disparities that warrant further investigation [32]. This pilot study provides insights into oral health behaviors and outcomes in DM patients compared to non-DM controls, highlighting significant differences in oral hygiene practices, self-reported dental conditions, and the impact of DM on oral health. A total of 99 DM patients and 102 controls participated, with DM patients exhibiting poorer oral health outcomes. Raising awareness about oral health can be a new tool for professionals in the prevention and management of DM. This finding emphasizes the need to include oral health education as an integral part of diabetes care programs.

Despite similar rates of brushing teeth twice a day (69.2% among PwD and controls), patients with DM had fewer teeth on average. Only 54.5% having 20 or more teeth compared to 70.6% of the control group. However, this relevant effect was found not significant in our statistical models. This finding aligns with existing literature, indicatings that individuals with DM are at higher risk of tooth loss due to the chronic inflammatory nature of periodontal disease commonly associated with DM [33, 34].

The reciprocal relation between DM (dysglycemia) and oral health indicates a detrimental cycle where one factor negatively influences the other. This interaction underscores the complexity of managing DM, where systemic inflammation, immune response, and oral microbial changes contribute to worsening oral health [11, 35]. Several diabetes associations, including the American Diabetes Association (ADA) and the International Diabetes Federation (IDF), recommend maintaining HbA1c levels below 7–8% to reduce the risk of complications [36]. In parallel, dental associations, such as the American Academy of Periodontology (AAP) and the European Federation of Periodontology (EFP), emphasize that individuals with diabetes should undergo regular dental check-ups (at least annually) and have their glycemic status monitored to minimize the risk of oral complications, including implant failure, impaired bone healing, delayed wound healing, and increased susceptibility to infections [37].

The relationship between DM type and oral health behaviours was particularly noteworthy. A higher proportion of patients with T1DM used advanced blood glucose monitoring technologies, such as sensors, and they generally reported better oral health compared to T2DM patients. T1DM patients were less likely to wear dentures, with 78.8% not using any form of dental prostheses, suggesting that their dental hygiene might be better. This is in contrast with T2DM patients, where self-reported dental and gum conditions were often rated as “average,” indicating possible neglect of oral health or the cumulative effect of older age and prolonged duration of the disease [38].

Interdisciplinary cooperation is needed to provide the best care for our patients. Integrating dental care with diabetes management can address the interconnected risks and improve overall patient outcomes.

It is noteworthy that the study found no significant effect of number of teeth on HbA1c levels when controlling for age and gender, indicating that the severity of metabolic disease, characterized by higher HbA1c, is not affected by the number of teeth. However, the broader impact of DM on oral health is likely to involve complex interactions between systemic inflammation, immune response, and oral microbial changes [32].

Oral hygiene practices, such as the frequency of tooth brushing and dental flossing, differed between DM and non-DM individuals. Although 69.2% of participants brushed their teeth at least twice daily, this rate was slightly higher among T2DM patients (71.2%) compared to T1DM patients (45.5%). However, despite frequent brushing, the DM group tended to have fewer teeth compared to the control group. This may be attributed to the cumulative effects of DM on oral tissues, impaired wound healing, and susceptibility to infections, factors that significantly increase the risk of tooth loss [32]. The results underscore the importance of integrating specific oral health education and preventive care into DM management programs.

The use of dental floss and interdental cleaning aids was less common among DM participants; only 17.2% of DM patients reported flossing, compared to 29.4% in the control group. This discrepancy suggests that patients with DM may benefit from additional education on comprehensive oral hygiene practices, beyond regular brushing.

The use of logistic regression models highlighted the complex interplay between diet, oral hygiene, and dental outcomes, emphasizing that higher fruit consumption and inadequate flossing may lead to a significantly increased risk of severe tooth loss, independent of DM status. These findings underscore the universal need for interdisciplinary care models that integrate oral health into diabetes management protocols [3].

A key limitation of this study is the reliance on self-reported data, which may not fully reflect clinical oral health status. Self-reported measures are subject to recall bias and individual perception differences, potentially affecting accuracy. Future research should integrate objective clinical assessments, such as dental examinations and periodontal evaluations, to validate these findings and provide a more comprehensive understanding of oral health outcomes in diabetes patients.

Overall, the study highlights the need for a multidisciplinary approach to DM care, emphasizing the integration of routine dental check-ups, improved education on oral hygiene, and close collaboration between diabetologists and dentists to address the oral health risks associated with diabetes.

### Limitations

As a pilot study, this research has inherent limitations, including a small sample size and reliance on self-reported data, which may introduce biases. Additionally, the cross-sectional design prevents causal inferences. Despite these constraints, the findings offer valuable preliminary insights and highlight the need for larger, longitudinal studies. This pilot study provides a foundation for future, larger-scale research to validate these preliminary findings and explore causal relationships in greater depth.

## Conclusion

The findings of this study highlight significant oral health disparities between DM and non-DM individuals. Patients with DM, particularly those with T2DM, report poorer oral health outcomes, more frequent denture use, and fewer natural teeth despite similar oral hygiene practices. These results emphasize the need for targeted oral health interventions, including comprehensive oral hygiene education, preventive care, and stronger integration between dental and diabetes care.

Annual dental check-ups are recommended for all DM patients, with biannual visits for those with periodontal disease. The “Budapest pilot” at the Semmelweis University Diabetes Dental Research Group further reinforces the importance of early referral and interdisciplinary cooperation to enhance patient outcomes. From a public health perspective, improving oral health awareness among DM patients is essential for prevention and long-term disease management. Future research will focus on conducting longitudinal studies to further explore the relationship between diabetes and oral health, as well as developing targeted preventive strategies.

## Electronic supplementary material

Below is the link to the electronic supplementary material.

Supplementary Material 1

## Data Availability

The datasets used and/or analyzed in the current study are available from the corresponding author upon reasonable request. These datasets include de-identified participant data and comply with ethical and privacy considerations.
