# Supplementary material for: Oral health practices and literacy in Hungarian diabetes patients: insights from a pilot-study using a WHO-adapted questionnaire
Source: BMC Oral Health. 2025 Mar 26;25:431. doi: 10.1186/s12903-025-05820-x (PMC11938766; doi:10.1186/s12903-025-05820-x)
Supplement: Supplementary file 1 — Supplementary Material 1 [file 12903_2025_5820_MOESM1_ESM.docx]

| Frequency of teeth cleaning | How often participants clean their teeth. |
| --- | --- |
|  | We binarized in two gruops:   Once a day   At least twice a day |
| Dental flossing | Using oral hygiene aids especially for dental floss. |
|  | Yes or no. |
| Regularity of dental visits | The time elapsed since the participant's last dental visit. |
|  |  Less than 6 months   6-12 months   More than 1 year but less than 2 years   2 years or more but less than 5 years   5 years or more   I have never visited a dentist |
| HbA1c level | The level of your last HbA1c. |
|  | HbA1c represents the average blood glucose level over the past three months and is regularly monitored during diabetology outpatient visits. In this study, participants self-reported their most recent HbA1c value through a specific question in the questionnaire. |
| Fruit and rapidly absorbable carbohydrate consumption | At least once a day compared to less frequent occurrences. |
| Dental status | The number of natural teeth a participant has. |
|  | The dental status aspect could only be assessed based on the number of teeth, as no clinical examination was performed. Dental status referred to the number of teeth. The number of teeth was categorized into four groups:   No natural teeth   1-9 teeth   10-19 teeth   20 or more teeth |
| Diagnosis of diabetes mellitus | Diabetes mellitus was defined based on clinical diagnosis, with no additional measurements performed to confirm diabetes status. |
